# Coping and Life Satisfaction in Colombian Older Adults

**DOI:** 10.3390/ijerph182010584

**Published:** 2021-10-09

**Authors:** Maria-Fernanda Reyes, Encarnación Satorres, Iraida Delhom, Alexandra Bueno-Pacheco, Juan C. Meléndez

**Affiliations:** 1 Faculty of Psychology, Universidad El Bosque, Av. Cra 9 No. 131 A-02, Bogota 110121, Colombia; reyesmariafr@unbosque.edu.co; 2 Department of Developmental and Educational Psychology, Faculty of Psychology, University of Valencia, Av. Blasco Ibañez 21, 46010 Valencia, Spain; Encarna.Satorres@uv.es; 3 Universidad Internacional de Valencia, Pintor Sorolla, 21, 46002 Valencia, Spain; idelhom@universidadviu.com; 4 Faculty of Philosophy and Educational Sciences, University of Azuay, Cuenca, Av. 24 de mayo 7-77, Cuenca 010107, Ecuador; abueno@uazuay.edu.ec

**Keywords:** coping, well-being, life satisfaction, aging, structural equation model

## Abstract

Background. Effective coping strategies facilitate older adults’ optimal adaptation and contribute to their well-being. Problem-focused strategies are associated with active styles and enhance well-being. This study analyzes the role of coping strategies in Colombian older adults’ subjective well-being (SWB) using structural equation modelling. Additionally, Confirmatory Factor Analyses of the Life Satisfaction Scale and Coping Strategies Questionnaires are performed. Method. A cross-sectional study is conducted with 455 Colombian older adults, ranging from 65 to 92 years old. Results. The results show that problem-focused coping has a positive effect on SWB, whereas emotion-focused coping has a negative effect on SWB. Conclusions. This article highlights the relationship between effective coping and life satisfaction by showing that problem-focused coping strategies are adaptative and enhance well-being during aging.

## 1. Introduction

One of the keys to maintaining well-being in aging is the use of effective coping strategies that facilitate older adults’ optimal adaptation to this stage. Knowing which strategies can enhance this process and lead to an increase in life satisfaction will help to establish some psychological and personal guidelines for successful aging and an important indicator of positive functioning of mental health, which is one of the main objectives of health policies in developed countries.

Subjective well-being (SWB) involves people’s appraisals and evaluations of their own lives [1]. It includes both reflective cognitive judgments, such as life satisfaction, and emotional responses to ongoing life, in terms of positive and pleasant emotions versus unpleasant and negative emotions [2]. Theory and empirical research suggest evidence for both stability and change in subjective well-being across the lifespan [3]. The study of SWB at different ages frequently characterizes it as a U-shaped curve that is the highest in youth, declining to its nadir in midlife, with an upswing after that [4]. This pattern holds for life satisfaction, positive emotions, and the lack of negative emotions [5]. Recently, the relationship between various measures of well-being and age in 145 countries has been examined, controlling for education and marital and employment status, among others, and confirmed the shape of the curve [6]. Research findings suggest that the older populations, although generally less healthy and less productive, might be more satisfied with their lives and experience less stress, worry, and anger than middle-aged people [7].

It is also important to examine coping trajectories of older adults later in life, as functional coping has a positive impact in mental health and wellbeing [8]. Coping is a set of cognitive and behavioral efforts and resources that the individual carries out to manage external and internal demands that the individual evaluates as stressful and exceed its control [9]. Based on this definition, coping has been classified into two essential domains: the first domain, problem-focused coping, refers to the individual’s efforts and resources to modify the problematic circumstances or the contribution of new resources that counteract the aversive effect of environmental conditions. The second domain, emotion-centered coping, refers to efforts and resources aimed at regulating distressing emotions. The purpose of coping strategies is to counterbalance, mitigate or alleviate stressful situations by reframing objectives or adjusting to a new and positively assessed situation [10]. Emotion-focused coping is considered a maladaptive strategy, because it is commonly reported to generate mental health issues, such as depression and anxiety [11], whereas problem-focused coping is consistently associated with positive health outcomes.

Older adults have to face multiple changes during aging that can be interpreted as stressful, and a favorable adjustment to these changes determines whether older adults can adapt optimally to aging, obtaining satisfaction. The type of coping strategies activated in different situations influences well-being and mental health [12].

Whereas several studies have found relationships between age, personality [13], social and economic factors [14], or physical health and SWB, the influence of coping strategies on SWB in older adults has not received much attention. Problem-focused strategies associated with active styles appear to enhance well-being; in contrast, emotion-focused approaches tend to be less successful [15,16]. The association between age and positive effect has been found to be fully mediated by problem-focused coping, and emphasized that using problem-focused coping strategies in stressful contexts and situations is beneficial for older adults [17]. In a predictive model [16], it has been found that both problem-centered and emotion-focused models were strongly related with subjective well-being, although the sign of the relationship was different, positive in the case of problem-oriented strategies. Problem-focused coping strategies would be facilitators of behavioral change because they involve problem-oriented actions rather than self-regulation [18].

Therefore, this study analyzes the role of coping strategies in older adults’ subjective well-being by testing structural equation models (SEM). The hypotheses state that: (a) problem-focused coping will have a positive effect on SWB; (b) emotion-focused coping will have a negative effect on SWB. Additionally, we analyze the construct validity of the scales by using a confirmatory factor analysis.

## 2. Materials and Methods

### 2.1. Participants

The sample was composed of 455 older adults in Colombia, from which 61% were women and 39% men, with ages between 65 and 92 years (*M =* 71.5, *SD* = 7.2), recruited in Bogotá, Colombia. Inclusion criteria were: 65 years and older and not being institutionalized. Exclusion criteria were: older adults with moderate to severe cognitive impairment and being institutionalized.

Regarding their marital status, 42.4% were married, 32% widowed, 13.2% single, and 12.4% divorced. Moreover, the majority of the sample (48.3%) had attended 3 to 5 years of elementary school, 22.3% reported having attended only middle school or high school, 7.0% had a technical degree, 11% had university studies, and 11.9% had never attended any academic institution.

### 2.2. Instruments

The Satisfaction with Life Scale (SWLS) was designed to assess the cognitive dimension of subjective well-being [19]. The questionnaire is aimed at measuring the global evaluation that individuals make about their satisfaction with life. The scale has five items with 7-point Likert response options that range from strongly agree (7) to strongly disagree (1). The SWLS has shown good construct validity and reliability; the alpha value in this study was 0.80.

The Coping Strategies Questionnaire (CSQ) is a 42-item self-report measure [20] designed to assess seven basic coping styles: (1) problem-solving coping, (2) negative auto-focused, (3) positive re-evaluation, (4) overt emotional expression, (5) avoidance, (6) seeking social support, and (7) religious coping. The questionnaire was validated for older adults through confirmatory factor analysis [21]. The test consists of two first-order correlated factors: problem-focused coping (problem-solving coping, positive re-evaluation and seeking social support) and emotion-focused coping (negative auto-focused, overt emotional expression, avoidance, and religious coping). The questionnaire has shown good psychometric properties. Cronbach’s alpha values for this study were: 0.81 problem-solving, 0.70 positive re-evaluation, 0.91 seeking social support, 0.65. negative auto-focused, 0.71 overt emotional expression, 0.70 avoidance, and 0.82 religion.

### 2.3. Procedure

Participants were contacted through “word of mouth” and community leaders in different sectors in Bogotá, Colombia. Initially, the older individuals were invited to participate. In this invitation, they were informed about the purpose of the study, the duration, and potential risks. It was emphasized that participation was voluntary and confidential, and that they could end their participation at any time. The older adults who were interested in participating and met the inclusion criteria gave their consent and filled out the scales with the support of a trained research assistant. When their participation ended, they were informed that if they were interested in the results, they could request them, and contact information was provided.

### 2.4. Statistical Analysis

First, correlations between coping strategies and life satisfaction were performed, followed by a Confirmatory Factor Analysis using the robust estimator maximum likelihood estimation (MLM) being performed using MPlus 7.3 (Muthén & Muthén, Los Angeles, CA, USA). MLM is a robust estimator of non-normality [22]. Second, a structural equational modelling technique with an MLM estimator was conducted. To assess the model’s fit, we used chi-square (*X*^2^) and incremental fit indices such as comparative fit index (CFI), Tucker-Lewis fit index (TLI) (≥0.90), and root-mean-square error of approximation (RMSEA) (≤0.07) [22].

## 3. Results

First, we performed bivariate correlations between the seven coping strategies and life satisfaction (see Table 1). The results showed significant and positive correlations between problem solving and positive re-evaluation and life satisfaction, and a negative association between negative auto-focused coping and life satisfaction. The coping strategies overt emotional expression, avoidance, (6) seeking social support, and (7) religious coping were not significantly associated with life satisfaction.

### 3.1. Confirmatory Factor Analysis (CFA)

We conducted CFAs for each scale using the robust estimator MLM. For the Life Satisfaction Scale (LS), we performed a first-order factor model, obtaining good fit (MLM*χ*^2^_(5)_ = 110.43 *p* < 0.001, CFI = 0.990, RMSEA = 0.04 90% CI (0.007–0.080)) and significant and adequate factor loadings. No modifications were needed.

For the Coping Strategies Questionnaire (CSQ), a second-order confirmatory factor analysis was performed. As originally proposed [20], the model was composed of seven first-order factors (problem-solving coping, negative auto-focused coping, positive re-evaluation, overt emotional expression, avoidance coping, social support seeking, and religious coping) and two second-order factors (problem-focused coping and emotion-focused coping). Problem-focused coping consists of problem-solving coping, positive re-evaluation, and social support seeking, whereas emotion-focused coping consists of negative auto-focused coping, overt emotional expression, avoidance coping, social support seeking, and religious coping. The second-order factors and factor loading path to 10.0, and the second-order factor variance and the residual variances associated with the first-order factors were constrained to equality [22].

The first model obtained an adequate fit for RMSEA, but a poor fit for CFI (see Table 1), and seven items showed non-significant factor loadings. We tested a second model without the seven non-significant items (9, 16, 24, 31, 39, and 40). The second model showed a good fit for all the fit indices (see Table 2). All the factor loadings and associations between the factors were significant.

### 3.2. Structural Model: Coping and Subjective Wellbeing

A structural equations model approach was used to test the associations between the latent factors of life satisfaction (LS) and the two-factor model for coping in a sample of Colombian older adults. The model was tested using the MLM estimator, which is a robust estimator of non-normality [22].

As the hypothesized model, we proposed that coping would be related to subjective well-being (SWB), and that the types of coping would be related differently to SWB. Thus, we expected a positive association between problem-focused coping and SWB and a negative association between emotion-focused coping and SWB.

**Figure 1:**
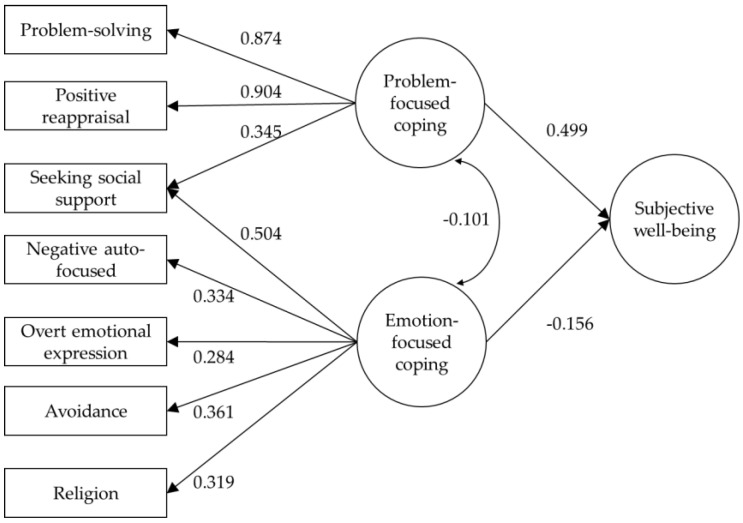
Final model.

The results for the hypothesized model showed a satisfactory fit: MLM*χ*^2^(731) = 1130.483, *p <* 0.001, MLMΔ*χ*^2^/df = 10.045, CFI = 0.901, RMSEA = 0.035, 90% CI (0.031–0.039). The results revealed significant associations between SWB and the two types of coping (see Figure 1). The findings showed that problem-focused coping had a positive and moderate association with SWB (*r* = 0.499, *p <* 0.001), whereas the relationship between emotion-focused coping and SWB was negative but weak (*r* = −0.156, *p* < 0.001).

## 4. Discussion

The current study analyzed the association among coping styles and subjective wellbeing in a group of Colombian older adults. First, the measurement models of the scales were assessed, showing that the Life Satisfaction Scale had an adequate model fit and providing evidence for the construct validity of the scale for the Colombian sample. Second, a second-order confirmatory factor analysis of the CSQ was performed. The results showed that a modified version of the second-order model proposed originally fit the data [20]. However, important modifications in the measurement model were performed, including the elimination of non-significant items. The modified version showed a satisfactory fit for a coping model with two second-order factors (problem-focused coping and emotion-focused coping). In addition, the coping strategy of seeking social support loaded in both coping factors, which was similar to the results reported in other research [12].

The structural part of the tested model offered evidence about the prediction of SWB in older adults and confirmed that problem- and emotion-focused coping were related to SWB. As hypothesized, a positive and moderate association was found between problem-focused coping and SWB, and a negative association between emotion-focused coping and SWB. Nonetheless, this latter relationship was weak. In addition, the results confirmed the idea that problem- and emotion-focused coping are complementary strategies, rather than two completely different and independent dimensions.

Subjective well-being and problem-focused coping are both key indicators of positive psychological functioning associated with mental health. Individuals who reported high SWB were at a lower risk of having a variety of psychological and maladaptive problems, and problem-focused strategies served to manage or alter the problem causing the distress; thus, facilitating adaptation. The results showed that the use of problem-oriented strategies facilitated the achievement of SWB. These results were similar to those obtained in older adults [12], who pointed out that this type of strategy was highly linked to well-being outcomes.

Problem-focused coping strategies are a strong predictor of good mental health outcomes [23]. Problem solving, as a coping strategy, has been found to be one of the most adaptive and active ways to face difficult situations. It has been related to improvements in quality of life [24], and it is useful for coping with negative emotions, anxiety, and dissatisfaction with life [25]. Reappraisal from a positive perspective promotes addressing problems proactively and, therefore, tends to decrease avoidance behavior and increase adaptation to change [26]. Furthermore, applying a positive reassessment strategy has been shown to be helpful in adjusting to health-related problems, which are typically associated with age [27]. Social isolation is a well-known problem for older adults, and it is associated with various negative health outcomes. Conversely, social support seems to have a larger direct effect on life satisfaction than objective aspects of social resources, such as network size and time spent with family members [28].

Emotion-focused coping involves individuals’ self-regulation to minimize the emotional consequences of stressful situations. Even though as maturity and refinement of emotional regulation increase with age, older adults can implement emotion-focused strategies very effectively because [29] a negative association was found between emotion-focused coping strategies and SWB. These types of strategies are more likely to be used when older adults are faced with uncontrollable stressors, the ones they believe they cannot handle, such as bereavement or serious health problems, situations highly associated with age. The results obtained are similar to previous research that obtained a significant and negative relationship between emotion-focused coping and SWB [16]; however, this research obtained showed a higher predictive power in the relationship between emotion-focused strategies and well-being. Functional coping strategies have been shown to positively affect the mental health of older adults [8].

In relation to specific strategies, the negative perception of oneself as unable to find solutions to stressful situations makes a person less likely to apply active solutions, reducing SWB. Furthermore, people who tend to repress their feelings and avoid offering active solutions to conflict situations tend to develop recurrent thought processes. The use of this strategy for a short period of time can be adaptive, but continuing to use it for a long time hinders the adaptation process [30]. Concerns about the consequences of expressing emotions in the presence of changes can decrease the positive regulation of emotions. Initially repressing them and using overly emotional expressions as a system of emotional overflow results in decreases in SWB due to regret about these expressions. Finally, the connection between religion and SWB remains unclear. Although religion is an emotion-oriented coping strategy, some authors point out that when subjects face situations of loss or negative changes without the possibility of personal control, religious strategies can be a way of effectively adapting to the situation. In general, religiosity is associated with benefits for physical and mental health [31]. However, the relationship between religion and well-being is not always positive because individuals use their faith and religion to cope with distress in different ways. Religious strategies are positive when they serve to find strength and relief, whereas negative religious coping reflects the conflict within oneself and facilitates the perception of negative life events as a form of punishment. Negative religious coping is a strong predictor of psychological suffering and is linked to higher levels of psychological distress [32].

The study also had some limitations. First, the study’s cross-sectional design did not allow us to draw strong inferences about causality. Consequently, the associations found in our study should be interpreted with caution. Second, some items were eliminated from the CSQ. However, we proposed a modified model that fits the data better, and we contributed a questionnaire with adequate psychometric properties that can be used to assess coping strategies in Colombian older adults.

This article highlights the relationship between effective coping and life satisfaction. Problem-focused coping strategies are adaptative and enhance well-being in aging. Subjective well-being and mental health are closely related, and the link could become increasingly important not only due to the increase in life expectancy, but also to the experience of loss situations that generate stress and older adults need to cope. Moreover, poor mental health is associated with lower SWB [33]. Research suggests that subjective well-being could be a protective factor for physical health by the risk of disease and promoting longevity [3]. Developing intervention strategies and promoting health policies to help older adults cultivate problem-oriented coping strategies will contribute to their life satisfaction and successful and healthy aging. The application of mindfulness or reminiscence therapy programs has been shown to be an effective intervention to promote adaptive strategies and increase life satisfaction in both healthy older adults and those with dementia [34,35].

## Figures and Tables

**Table 1:** Bivariate correlation coefficients between coping strategies and life satisfaction.

Coping Strategies	Life Satisfaction
Problem-solving coping	0.281 **
Negative auto-focused	−0.116 *
Positive re-evaluation	0.222 **
Overt emotional expression	−0.092
Avoidance	0.034
Seeking social support	0.013
Religious coping	−0.005

Note. * *p* < 0.050, ** *p* < 0.001.

**Table 2:** Fit indices for second-order factor models for CSQ.

Model	*χ* ^2^	*gl*	Δ*χ*^2^	Δ*gl*	CFI	RMSEA	RMSEA IC 90%
CSQ Model 1	2297.71 *	770	1476 **		0.67	0.06	0.06–0.07
CSQ Model 2	820.86 **	551		219	0.92	0.03	0.03–0.037

Note. * *p* < 0.050, ** *p* < 0.001.

## Data Availability

The data presented in this study are available on request to the authors. The data are not publicly available due to privacy reasons.
